# Altered Brain Functional Connectivity in Small-Cell Lung Cancer Patients after Chemotherapy Treatment: A Resting-State fMRI Study

**DOI:** 10.1155/2017/1403940

**Published:** 2017-07-17

**Authors:** Konstantinos Bromis, Kostakis Gkiatis, Irene Karanasiou, George Matsopoulos, Eustratios Karavasilis, Matilda Papathanasiou, Efstathios Efstathopoulos, Nikolaos Kelekis, Vasileios Kouloulias

**Affiliations:** ^1^School of Electrical and Computer Engineering, National Technical University of Athens, Athens, Greece; ^2^Hellenic Military University, Vari, Athens, Greece; ^3^Research Centre of Radiology and Imaging, “Evgenidion” General Hospital, Athens, Greece; ^4^2nd Department of Radiology, Radiotherapy Unit, ATTIKON University Hospital, Athens, Greece

## Abstract

Previous studies in small-cell lung cancer (SCLC) patients have mainly focused on exploring neurocognitive deficits associated with prophylactic cranial irradiation (PCI). Little is known about functional brain alterations that might occur due to chemotherapy treatment in this population before PCI is administered. For this reason, we used resting-state functional Magnetic Resonance Imaging (fMRI) to examine potential functional connectivity disruptions in brain networks, including the Default Mode Network (DMN), the Sensorimotor Network, and the Task-Positive Network (TPN). Nineteen SCLC patients after platinum-based chemotherapy treatment and thirteen controls were recruited in the current study. ROI-to-ROI and Seed-to-Voxel analyses were carried out and revealed functional connectivity deficits in patients within all the networks investigated demonstrating the possible negative effect of chemotherapy in cognitive functions in SCLC populations.

## 1. Introduction

Small-cell lung cancer (SCLC) is an aggressive subtype of lung cancer. About 10–15% of lung cancers are small-cell lung cancers. The standard therapy for patients suffering SCLC includes mainly two procedures: (1) platinum-based chemotherapy and (2) thoracic radiotherapy [1]. However, even with this therapeutic schema, this type of cancer is difficult to be treated due to its metastatic tendency, especially in the brain. A systematic review [2] showed that without PCI intervention, approximately 46% of SCLC population will eventually develop metastases in the brain within one year of diagnosis.

Prophylactic cranial irradiation (PCI) is a technique that has been shown to reduce the recurrence of brain metastases and improve overall survival in patients who responded to chemotherapy [3, 4]. Despite the absolute benefit of PCI in prolonging survival, there is evidence supporting its association in the development of cognitive deficits due to its neurotoxic effects in the brain [5]. Two recent longitudinal studies by Simó and her colleagues [6, 7] showed that neuropsychological deficits in SCLC patients treated with PCI are associated with PCI therapy, possibly in conjunction with platinum-based chemotherapy.

Little is known about functional brain alterations that might follow SCLC chemotherapy treatment. Four SCLC studies [8–11] which conducted neurocognitive assessments found evidence of impaired executive functioning, memory retrieval, and motor coordination in SCLC patients before PCI and after chemotherapy. Furthermore, an early study [12] revealed cognitive deficits in a non-small-cell lung cancer population right after chemotherapy treatment. Similarly, cognitive changes such as attention deficits [13–15] and executive functioning impairments [16, 17] have been reported in chemotherapy treated breast cancer populations.

Also, more recent neuroimaging techniques (Magnetic Resonance Imaging (MRI); Diffusion Tensor Imaging (DTI)) have been conducted in order to explore structural alterations related to chemotherapy treatment in cancer populations. Specifically, two studies [6, 11] found gray and white matter deficits in SCLC patients. Similar gray and white matter alterations were reported in a recent review of 17 structural MRI and DTI studies focusing on women with breast cancer treated with adjuvant chemotherapy [18].

In recent years, researchers have used the resting-state fMRI (rs-fMRI) method in order to examine the way chemotherapy treatment affects whole brain functional connectivity. RS-fMRI is a noninvasive technique measuring temporal interactions between brain regions when no task is performed [19]. Previous rs-fMRI studies have reported the existence of distinct functionally linked resting-state networks [20, 21]. Resting-state network analysis has been extensively used to examine functional connectivity across various clinical populations such as Alzheimer's disease, schizophrenia, and autism [22–24], demonstrating that cognitive impairments may be associated with connectivity disruptions in those networks.

Thus far, to the best of our knowledge the use of rs-fMRI to examine the underlying mechanisms of the potential cognitive deficits related to chemotherapy in SCLC patients has not been investigated yet. However, there is evidence of connectivity disruptions associated with cancer and chemotherapy in a significant number of functional connectivity studies. The majority of those studies [25–27] have focused on chemotherapy treated breast cancer patients reporting functional connectivity alterations in the Default Mode Network (DMN).

DMN is one of the most investigated resting-state brain networks which is activated when someone is not performing an explicit task and the brain is at wakeful rest or when it is performing internal tasks such as thinking about others and thinking about the past [28]. Brain regions involved in the DMN are the posterior cingulate cortex (PCC), medial prefrontal cortex (MPFC), lateral temporal cortex, and precuneus.

Kesler et al. [26] found disrupted DMN connectivity and used these results to accurately discriminate breast cancer patients from healthy subjects. Additionally, Simó and her colleagues [29] reported for the first time DMN functional connectivity abnormalities in lung cancer patients.

Except DMN, researchers have also investigated its anticorrelated network, the Task-Positive Network (TPN) [30] which is involved in cognitive processing functions such as attention and executive function as well as working memory. Brain regions related to TPN include the bilateral anterior intraparietal sulcus (AIPS), bilateral inferior parietal lobule, insula, middle temporal gyrus, supplementary motor area (SMA), and frontal eye field [30]. An fMRI study examining the effects of chemotherapy on functional connectivity in breast cancer population [25] reported disruptions within TPN suggesting the negative effects of chemotherapy regarding this network. In addition, a previous review of 14 fMRI studies in noncentral nervous system cancer populations [31] showed reduced activation in brain regions involved in executive function and episodic memory.

In this scientific and research milieu the current study aimed to examine the effects of chemotherapy on functional connectivity in SCLC patients. Since previous neuroimaging studies [25–27, 31] have suggested alterations after chemotherapy in functions associated with the DMN and TPN, we hypothesized that both these networks could be functionally disrupted in the SCLC population. Additionally, based on evidence from neurocognitive assessments for impaired motor coordination in SCLC patients, we examined the Sensorimotor Network (SMN), which involves various brain structures, including the premotor cortex, primary motor cortex, and primary somatosensory cortex, for potential alterations that may explain the underlying processes following these motor-specific complaints. To the best of our knowledge, the present study is the first attempt to examine functional connectivity disruptions following chemotherapy treatment in SCLC population.

## 2. Methods

### 2.1. MRI Acquisition

Whole brain MRI and fMRI data were collected on a Philips 3.0T scanner (Achieva; Philips, Best, The Netherlands) at the Radiology Research Unity, Medical Imaging Department, Evgenidion Hospital, National and Kapodistrian University, Athens, Greece, using an 8-channel SENSE head coil. Foam pads and headphones were used to reduce head motion and scanner noise.

### 2.2. Volumetric Sequences

Anatomical imaging was performed with T1 weighted 3D sagittal acquisition (1.0 mm thick slices, 0 mm slice gap, TE = 4.6 msec/TR = 15 msec, FOV = 256, and 1.0 × 1.0 × 1.0 mm reconstructed voxel size).

### 2.3. Rs-fMRI Sequence

Functional MRI data were acquired while subjects were lying quietly in the scanner with eyes closed. Whole brain rs-fMRI was performed using a gradient echo planar imaging sequence (TR = 2000 msec/TE = 30 msec, flip angle = 90, and 3.0 × 3.0 × 3.0 reconstructed voxel size). For maximum consistency, all subjects were instructed to close their eyes throughout the rs-fMRI sequence, relax, but to remain awake and motionless as much as possible during the data acquisition.

### 2.4. Participants

Fourteen healthy participants and twenty-four SCLC patients after chemotherapy and before PCI treatment volunteered in the current study. All participants were native Greek speakers and right handed (self-reported), met the standard MRI safety criteria, and had no history of diagnosed neurological disorder or major psychiatric disorder. A summary for both cohorts and a summary of the cancer stage and regimens are presented in Table 1. Apart from smoking history (*p* < 0.002) there were no statistical significant differences between the two groups. All participants provided written informed consent and the study was approved by the appropriate research ethics committee.

Exclusion criteria for participants were as follows: (i) motion artifacts that were above a certain threshold (2 mm), (ii) presence of brain metastatic tumor, and (iii) any treatment with psychotropic medication including substance misuse. Based on these criteria, one healthy subject and four patients were excluded due to excessive motion. Furthermore, one patient was excluded due to brain metastasis at the time of MRI acquisition. At the end, data from thirteen controls and nineteen SCLC patients met the inclusion criteria to proceed to further analysis.

### 2.5. Preprocessing

Data were preprocessed using FMRIB's Software Library (v. 5.0.7; https://www.fMRIb.ox.ac.uk/fsl) [32]. After reorienting, anatomical images were manually skull-stripped using FSL's brain extraction toolbox [33]. Functional data were motion corrected (MCFLIRT) and spatially smoothed using a Gaussian kernel of 5 mm full width at half maximum and grand-mean scaled to ensure that the statistical implementation will be carried out properly [34]. Registration has been implemented in two steps. In the first step, each subject's 4D dataset was aligned to each brain extracted T1 anatomical image using the Boundary Based Registration (BBR) method [35]. This method was applied instead of the widely used rigid body transformations (6 DOFs) because it has been shown to produce more accurate and robust results. In the second step, each of these new datasets was registered to a template (MNI152, 2 mm resampling resolution) using both linear and nonlinear transformations in order to create a common space for statistical analysis.

### 2.6. Functional Connectivity Analysis

Functional connectivity analysis has been conducted using Conn, a Matlab based functional connectivity toolbox [36]. In order to increase the reliability of the resting-state fMRI data and reduce noise, additional preprocessing steps were performed. Specifically, the CompCor approach was implemented, a method that extracts five principal components from white matter and five from CSF time series. White matter and CSF were derived via segmentation of each subject's anatomical images. Each of these components was included as confounds in the denoising step of Conn [37]. In addition, the six motion parameters that were extracted from the preprocessing steps as well as a regressor matrix having time points that were corrupted by large motion (fsl_motion_outliers script-Round Middle Square Intensity Difference as metric) were also added as confound factors and linearly regressed out in the aforementioned procedure. Afterwards, a band pass filter with a frequency window [0.01–0.1 Hz] was applied in order to enhance retest reliability [38].

#### 2.6.1. Target Regions of Interest

The regions of interest that have been used were provided by the Functional Imaging in Neuropsychiatric Disorders Lab of the Stanford University [39, 40]. The selection of the above ROIs was based on their cluster size. Their small size allows investigating functional connectivity in different brain regions while not being susceptible to noise.

Dorsal-DMN and Ventral-DMN (34 regions), together determining the DMN, Sensory-Motor Network (SMN) (13 regions) and Task-Positive Network (TPN) (40 regions), were identified and passed in the first and second-level analyses.

#### 2.6.2. Seed Regions

Three ROIs that together comprise the bilateral posterior cingulate cortex (PCC) were determined as seed regions to investigate the DMN. PCC is known to be a central functional hub of the DMN retaining strong correlations with all the other regions of this network [41].

For the SMN, after implementing a sensitivity analysis using every bilateral pair of the predefined ROIs of the sensory-motor network as seeds, the left and right primary motor cortices were found to have the highest correlation with the other motor regions and were used as a seeds in the current analyses.

For the TPN, the bilateral intraparietal sulcus regions were defined as seed regions based on previous studies on external attention and working memory [30, 42, 43].

#### 2.6.3. First Level Analysis

Seed-to-Voxel and ROI-to-ROI full correlation functional connectivity maps (hrf weighted) were created for each subject. Specifically, for the Seed-to-Voxel analysis *r*-Pearson correlation coefficients between each seed and the rest of the brain voxels were computed. For the ROI-to-ROI analysis, the mean BOLD time series from each ROI and *r*-Pearson correlation coefficients, between each seed ROI and each target ROI, were calculated. A Fisher *r*-to-*z* transformation was performed in both analyses in order to efficiently implement general linear models in the second-level analysis.

#### 2.6.4. Second-Level Analysis

Seed-to-Voxel connectivity maps and ROI-to-ROI connectivity matrices were computed for each participant and entered in the second-level analysis. Age and sex were entered as covariates of no interest. Contrasts between sources were selected in order to account for the main effect of each seed region in the analyses.

*Seed-to-Voxel*. Within group Seed-to-Voxel analysis was performed to demonstrate the occurrence of the investigated networks. A peak voxel intensity threshold of *p* < 0.000001 uncorrected and a threshold of *p* < 0.05 FDR corrected for cluster size was set for a two-sided contrast (positive and negative correlations) analysis.

Afterwards, a between group analysis was conducted to examine functional connectivity changes between the control and patient groups. The threshold for significant changes was set to *p* < 0.01 for peak voxel intensity and to *p* < 0.05 FDR corrected for cluster size. 

*ROI-to-ROI*. For the ROI-to-ROI analyses, each network was investigated separately. In within group analyses, the threshold was set to *p* < 0.05 FDR corrected. In between group analyses the threshold was set to *p* < 0.05 FDR corrected.

## 3. Results

### 3.1. Within Group Functional Connectivity

**Figure 1:**
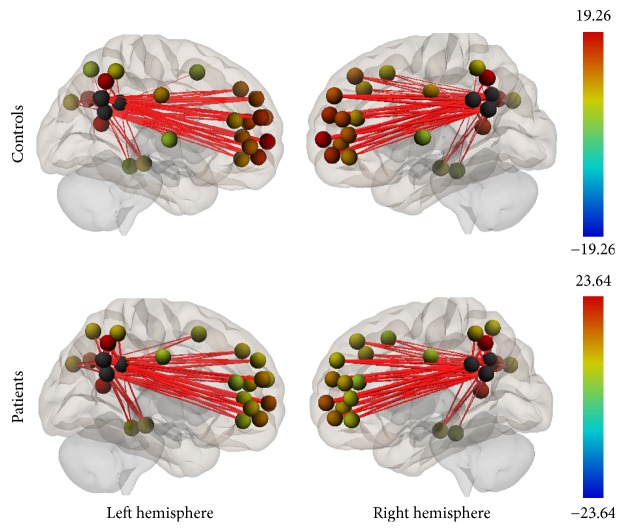
Functional connectivity map of the ROI-to-ROI analysis for the DMN (*p*-FDR < 0.05). Line thickness corresponds to the actual *p* value of the connection. The color of the balls denoting the ROIs signifies the *p* value of the connection according to the colorbar.

**Figure 2:**
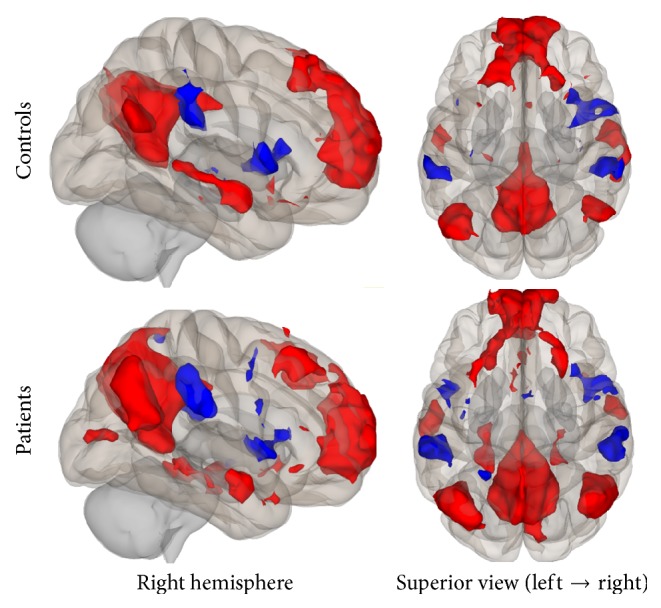
Functional connectivity map of the Seed-to-Voxel analysis for the DMN (*p*-unc < 0.0001; cluster *p*-FDR < 0.05). Red color indicates positive correlation while blue color indicates negative correlation.

**Figure 3:**
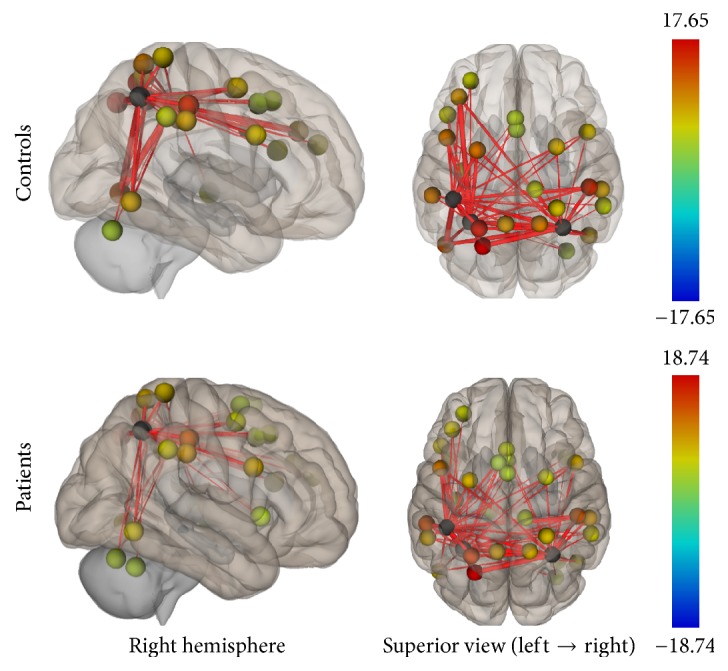
Functional connectivity map of the ROI-to-ROI analysis for the TPN (*p*-FDR < 0.05). Line thickness corresponds to the actual *p* value of the connection. The color of the balls denoting the ROIs signifies the *p* value of the connection according to the colorbar.

**Figure 4:**
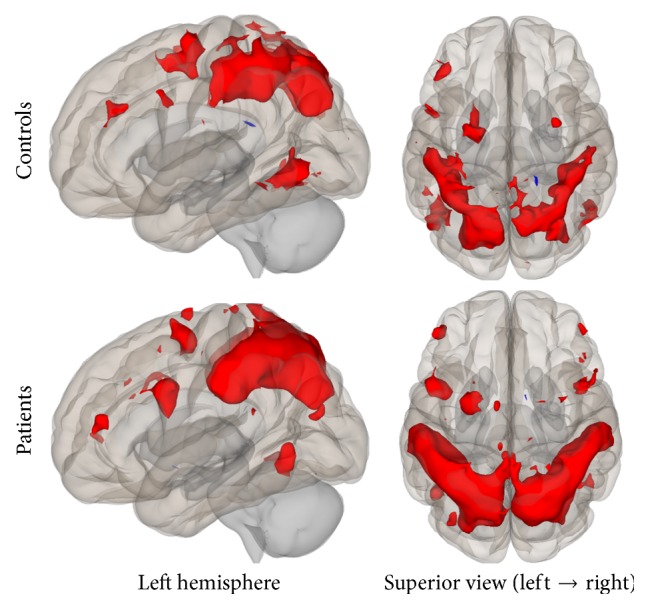
Functional connectivity map of the Seed-to-Voxel analysis for the TPN (*p*-unc < 0.0001; cluster *p*-FDR < 0.05). Red color indicates positive correlation while blue color indicates negative correlation.

**Figure 5:**
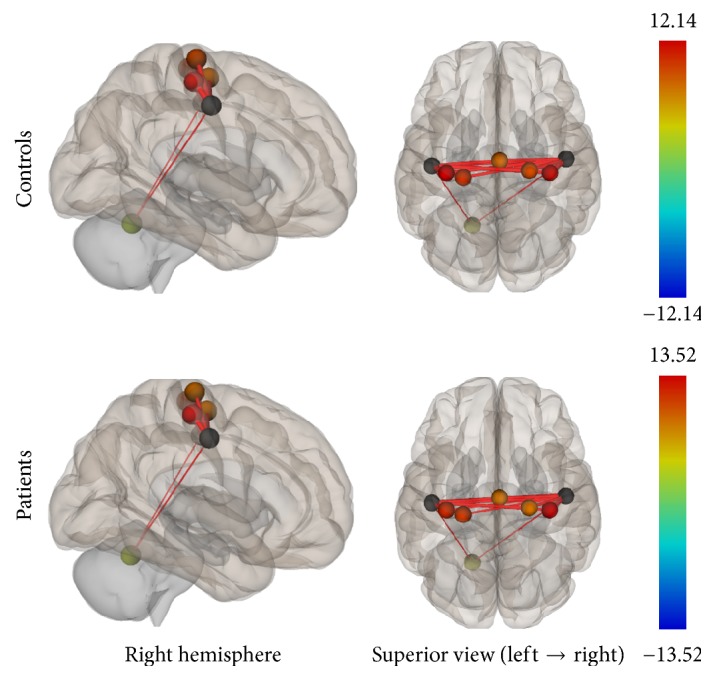
Functional connectivity map of the ROI-to-ROI analysis for the SMN (*p*-FDR < 0.05). Line thickness corresponds to the actual *p* value of the connection. The color of the balls denoting the ROIs signifies the *p* value of the connection according to the colorbar.

**Figure 6:**
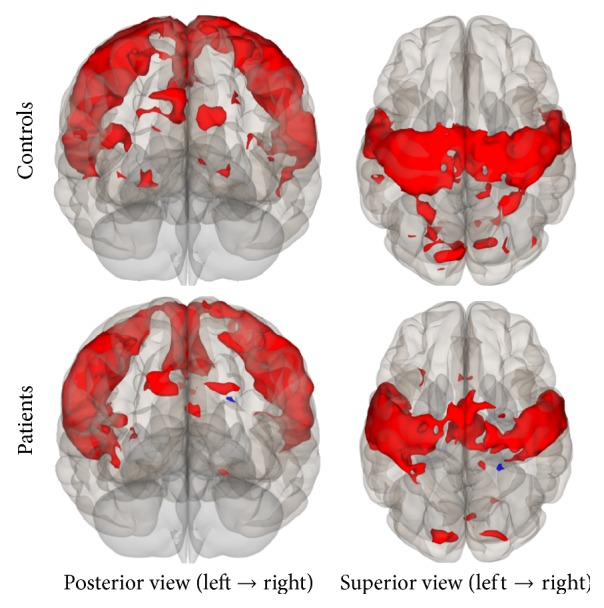
Functional connectivity map of the Seed-to-Voxel analysis for the SMN (*p*-unc < 0.0001; cluster *p*-FDR < 0.05). Red color indicates positive correlation while blue color indicates negative correlation.

Group connectivity maps of the DMN, TPN, and SMN were created based on the main effect of each seed region and were consistent in both ROI-to-ROI and Seed-to-Voxel analyses in the two groups (see Figures 1–6).

### 3.2. Between Groups Functional Connectivity

Between groups comparisons revealed lower functional connectivity for the patient group in all three networks that were investigated. In Tables 2 and 3 a summary of all the statistical significant results is presented for both analyses in all networks.

#### 3.2.1. Default Mode Network

**Figure 7:**
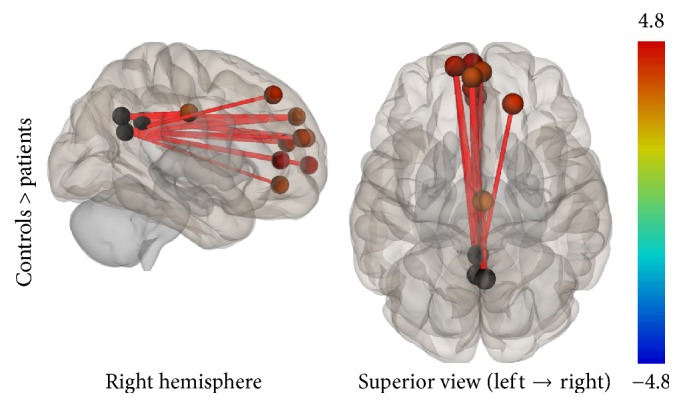
ROI-to-ROI analysis. Statistically significant lower functional connectivity in the patients compared to controls for the DMN (*p*-FDR < 0.05). Line thickness corresponds to the actual *p* value of the difference between the two groups. The color of the balls denoting the ROIs signifies the *p* value of the difference between the two groups according to the colorbar.

**Figure 8:**
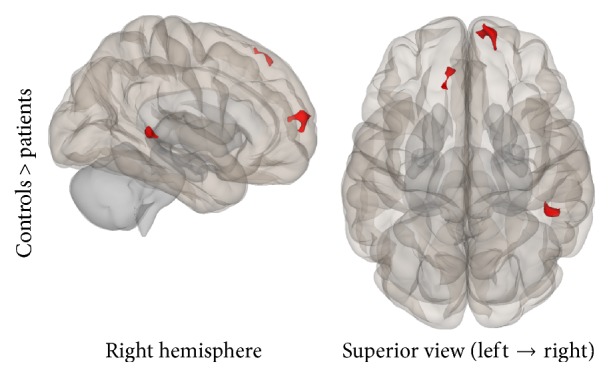
Seed-to-Voxel analysis. Statistically significant lower functional connectivity in the patients compared to controls for the DMN (voxel *p*-unc < 0.01; cluster *p*-FDR < 0.05). Red color indicates cluster with decreased functional connectivity for the patients.

The DMN connectivity map for the ROI-to-ROI analysis showed lower connectivity in the patient group between PCC (seed region) and the frontal pole, paracingulate gyrus, superior frontal gyrus, and cingulum. In the Seed-to-Voxel analysis, right frontal pole, right middle temporal gyrus, and left superior frontal gyrus were less correlated with the PCC in patients compared to controls (Figures 7 and 8).

#### 3.2.2. Task-Positive Network

**Figure 9:**
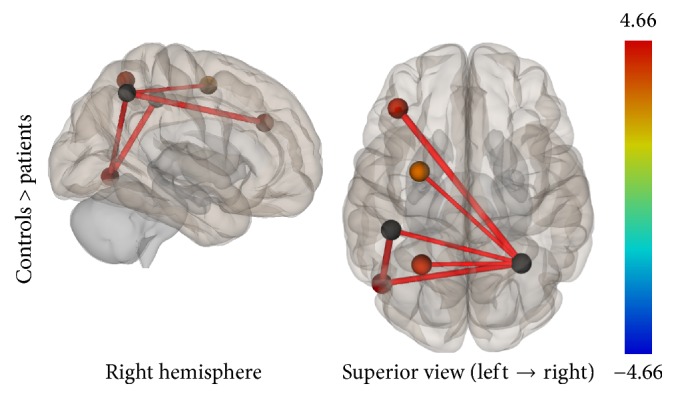
ROI-to-ROI analysis. Statistically significant lower functional connectivity in the patients compared to controls for the TPN (*p*-FDR < 0.05). Line thickness corresponds to the actual *p* value of the difference between the two groups. The color of the balls denoting the ROIs signifies the *p* value of the difference between the two groups according to the colorbar.

**Figure 10:**
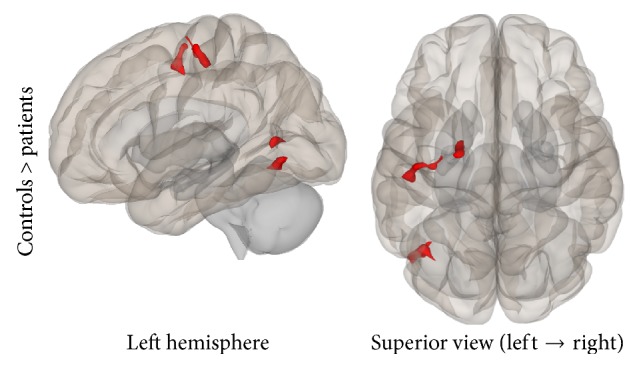
Seed-to-Voxel analysis. Statistically significant lower functional connectivity in the patients compared to controls for the TPN (voxel *p*-unc < 0.01; cluster *p*-FDR < 0.05). Red color indicates cluster with decreased functional connectivity for the patients.

In the ROI-to-ROI analysis, correlations between the AIPS (seed region) and the middle frontal gyrus, the visual cortex, the superior parietal lobule, and the premotor cortex all in the left hemisphere of the brain showed decrement in connectivity in the patient group compared to controls. In the Seed-to-Voxel analysis, the AIPS revealed lower connectivity in the premotor and primary somatosensory cortices, the superior frontal gyrus, and the lateral occipital cortex in the patients compared to controls (Figures 9 and 10).

#### 3.2.3. Sensorimotor Network

**Figure 11:**
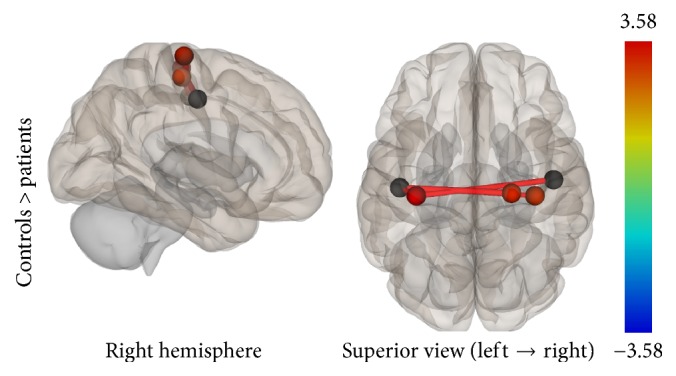
ROI-to-ROI analysis. Statistically significant lower functional connectivity in the patients compared to controls for the SMN (*p*-FDR < 0.05). Line thickness corresponds to the actual *p* value of the difference between the two groups. The color of the balls denoting the ROIs signifies the *p* value of the difference between the two groups according to the colorbar.

**Figure 12:**
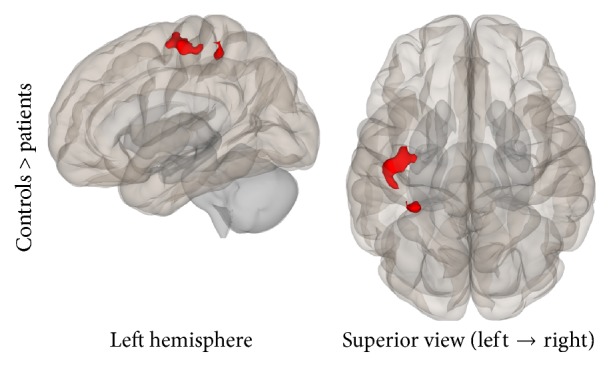
Seed-to-Voxel analysis. Statistically significant lower functional connectivity in the patients compared to controls for the SMN (voxel *p*-unc < 0.01; cluster *p*-FDR < 0.05). Red color indicates cluster with decreased functional connectivity for the patients.

Results from both analyses showed lower connectivity in the patient group compared to controls. Specifically, in the ROI-to-ROI analysis, the PSC (BA3b, seed region) was statistically significant less connected with the bilateral premotor cortex and the right primary motor cortex in the patient group. In the Seed-to-Voxel analysis, lower functional connectivity was found for the patients between the PSC and the left superior parietal lobule, left premotor, and primary somatosensory cortices (Figures 11 and 12).

## 4. Discussion

It is known from previous neuropsychological studies that neurocognitive processes, such as executive functioning, motor coordination, and working memory, are impaired in SCLC patients. The underlying sources of these impairments have caused contradictory research findings. The majority of the literature supports that these cognitive deficits are associated with PCI treatment [5–7]. However, there is not enough evidence that these impairments are not related to other sources, such as chemotherapy or cancer itself [8–11].

For this reason, the current study documents brain functional connectivity alterations in SCLC population after chemotherapy treatment and before undertaking PCI treatment. Based on previous neuroimaging findings in breast cancer [25–27] and lung cancer [29] populations as well as neurocognitive assessments in SCLC patients [8–11], we investigated chemotherapy-related connectivity disruptions within the DMN, TPN, and SMN. Following our hypothesis, SCLC group exhibited significantly lower connectivity within all the networks being explored.

DMN is one of the most examined resting-state brain networks correlated with many different functions, including autobiographical information, episodic memory, past retrieval, and other internal thoughts. We found disrupted connectivity within the DMN, particularly between the PCC (seed region) and regions of the prefrontal cortex including frontal pole, paracingulate gyrus, and superior temporal gyrus as well as the middle temporal gyrus and the cingulum.

Our findings are in line with previous neuroimaging studies in cancer populations, showing deficits in global DMN connectivity. Particularly, abnormalities in the PCC and the precuneus were the most consistent findings across breast cancer functional connectivity studies [25, 26, 44, 45] and one recent lung cancer rs-fMRI study [29]. Furthermore, two studies [29, 44] found a decrement in connectivity in the middle temporal gyrus. We observed a similar pattern of functional connectivity alterations in this brain region.

A review of task-based fMRI studies in breast cancer population [31] indicated, as the most consistent finding, hypoactivations in prefrontal and parietal regions. In addition, Kesler and colleagues [26] conducted a multivariate pattern analysis (MVPA) of DMN functional connectivity in order to distinguish chemotherapy treated survivors from healthy controls. Connectivity between DMN and prefrontal regions was the most distinct variable to separate the two groups providing 90-91% accuracy. These findings are in line with our report showing a consistency in abnormal connectivity between PCC and prefrontal cortex.

In addition to this, Inagaki et al. [46] reported smaller gray matter and white matter in prefrontal, cingulate gyrus, and precuneus one year after chemotherapy treatment in breast cancer population. Likewise, Kesler and colleagues [47] conducted graph theory analysis and found structural clustering alterations in right inferior and middle frontal gyri, bilateral postcentral gyri, right precuneus, and left inferior temporal gyrus in breast cancer patients, indicating that decrements in brain structure may affect functional connectivity.

Concerning TPN and chemotherapy, connectivity alterations within this network may provide an insight on how chemotherapy affects cognitive functioning. Regions included in TPN have been shown to be involved in attention-demanding tasks, executive function, and working memory. Previous neurocognitive assessments in SCLC population [8–10] have reported impaired frontal lobe executive functioning, learning, and working memory after chemotherapy treatment.

Following this, using intraparietal sulcus as a seed to trigger TPN, we found less connectivity in the middle and superior frontal gyrus, visual cortex, parietal lobule, premotor cortex, and primary somatosensory cortex. Similar patterns of functional connectivity disruptions were found in a previous task-based fMRI study [25] in breast cancer population. Authors reported differences in connectivity in premotor and visual cortex one month after completion of chemotherapy. However, these disruptions were partially recovered at one year.

As our patient group participated in the current study right after chemotherapy treatment, an appropriate subject group for future studies would be to examine if these specific disruptions are still present for longer periods of time after chemotherapy in patients that have or have not been treated with PCI, in order to evaluate the origins of these cognitive impairments in the SCLC population.

Regarding the literature, this is the first attempt through lung cancer population to examine potential functional connectivity alterations in the Somatosensory Network. Previous neurocognitive assessments have acknowledged the presence of fine motor coordination impairments in SCLC patients. Using primary somatosensory cortex as a seed, we reported significant decrements in connectivity in bilateral premotor cortex, right primary motor cortex, left primary somatosensory cortex, and left superior parietal lobule. Results have showed less interhemispheric functional connectivity between motor and sensory areas which may indicate a potential correlation with motor impairments. Future studies should evaluate this by examining correlations between functional connectivity and neuropsychometric tests.

Limitations of our study mainly include sample size and cognitive neuropsychological assessment. The sample size between the two groups is not balanced which may affect between group comparisons. The lack of information regarding cognitive performance of the patient group did not allow us to run a correlation analysis between functional connectivity and performance measures. Future studies should evaluate this in order to delineate how functional connectivity disruptions affect cognitive performance. Finally, the current design of our study has limited the possibility of examining if these alterations are specific to chemotherapy or may occur prior to this, possibly by cancer itself. A future perspective study should examine this, in order to isolate the effects of chemotherapy.

## 5. Conclusion

To the best of our knowledge, this is the first study to examine potential functional connectivity alterations in an SCLC population using rs-fMRI. Our findings suggest significant functional connectivity disruptions within all the networks investigated, demonstrating the possible negative impact of chemotherapy in cognitive functions in SCLC population.

## Figures and Tables

**Table 1:** Summary of demographics of the two groups. Statistical analysis (two-group, one-tailed, and Student's *t*-test) is performed between the two groups. Cancer stages and regimens for the patient group are presented as well.

	Controls (*n* = 13)	Patients (*n* = 19)	*p* value
Age (years)	56.38 (7.63)	54.78 (5.98)	0.49
Education (years)	17 (6.44)	13.31 (4.9)	0.07
Gender			
Male	9 (69%)	15 (79%)	0.53
Female	4 (31%)	4 (21%)	
Smoking	2 (14%)	12 (63%)	0.002
Stage	—	IIB – 9 (47%)	
	—	IIIA – 8 (42%)	
	—	IIIB – 2 (11%)	
Regimen_1^*∗*^		14 (74%)	
Regimen_2^*∗∗*^		5 (26%)	

^*∗*^Regimen_1: Cisplatin 60–80 mg/m^2^ day 1 + Etoposide 100–120 mg/m^2^ days 1–3 (every 21 days); ^*∗∗*^Regimen_2: Carboplatin 5 AUC day 1 + Etoposide 100–120 mg/m^2^ days 1–3 (every 21 days).

**Table 2:** ROI-to-ROI analysis. Statistically significant lower functional connectivity in patient group compared to controls.

Network	Seed regions	Target regions	Coordinates^*∗*^	*T*(30)	*p*-unc	*p*-FDR
Default Mode Network	Posterior cingulate cortex, bilateral	Superior frontal gyrus, left	(−4, 53, 33)	4.8	0.0001	0.0014
		Paracingulate gyrus, bilateral (BA32)	(−2, 42, −5)	4.15	0.0003	0.0028
		Frontal pole, left	(−13, 59, 20)	4.14	0.0003	0.0028
		Frontal pole, right	(−4, 60, 2)	3.36	0.0021	0.0176
		Cingulum, bilateral	(2, −16, 36)	3.33	0.0023	0.0255
		Superior frontal gyrus, right	(18, 38, 47)	2.97	0.0058	0.0319
		Superior frontal gyrus, bilateral	(1, 56, 19)	2.83	0.0082	0.0388
		Paracingulate gyrus, bilateral (BA9)	(−4, 47, 17)	2.77	0.0096	0.0394
		Paracingulate gyrus, bilateral (BA24)	(−2, 42, −10)	2.63	0.0133	0.0489

Task-Positive Network	Anterior intraparietal sulcus, bilateral	Middle frontal gyrus, left	(−40, 35, 29)	4.66	0.0001	0.0024
		Visual cortex-V5, left	(−49, −66, −6)	4.6	0.0001	0.0028
		AIPS^*∗∗*^, left (BA7)	(−44, −36, 45)	3.14	0.0037	0.0468
		Superior parietal lobule, left	(−26, −56, 57)	2.98	0.0056	0.0468
		Premotor cortex, left	(−28, −2, 53)	2.96	0.006	0.0468

Somatosensory Network	Primary somatosensory cortex, bilateral	Premotor cortex, right	(23, −21, 71)	3.58	0.0012	0.0132
		Premotor cortex, left	(−36, −22, 64)	2.89	0.0071	0.0392
		Primary motor cortex, right	(37, −22, 58)	2.62	0.0135	0.0497

^*∗*^mm: Talairach space. ^*∗∗*^Anterior intraparietal sulcus. Difference corresponds to the correlation between the two seed regions.

**Table 3:** Seed-to-Voxel analysis. Statistically significant lower functional connectivity in patient group compared to controls.

Network	Seed regions	Target region	Coordinates^*∗*^	Cluster size	Cluster *p*-FDR
Default Mode Network	Posterior cingulate cortex, bilateral	Frontal pole, right	(14, 62, 16)	96	0.016
		Middle temporal gyrus, right	(46, −40, 00)	74	0.029
		Superior frontal gyrus, left	(−08, 40, 48)	61	0.043

Task-Positive Network	Anterior intraparietal sulcus, bilateral	Premotor/primary somatosensory cortex, left	(−48, −22, 52)	128	0.004
		Superior frontal gyrus, left	(−20, −08, 62)	102	0.008
		Lateral occipital cortex, inferior division, left	(−42, −62, −08)	83	0.015
		Lateral occipital cortex, left	(−42, −62, 04)	61	0.044

Somatosensory Network	Primary somatosensory cortex, bilateral	Premotor/primary somatosensory cortex, left	(−48, −16, 58)	227	<0.001
		Superior parietal lobule, left	(−32, −40, 54)	76	0.041

^*∗*^mm: Talairach space.
